# Random Walks on Networks with Centrality-Based Stochastic Resetting

**DOI:** 10.3390/e25020293

**Published:** 2023-02-04

**Authors:** Kiril Zelenkovski, Trifce Sandev, Ralf Metzler, Ljupco Kocarev, Lasko Basnarkov

**Affiliations:** 1 Research Center for Computer Science and Information Technologies, Macedonian Academy of Sciences and Arts, Bul. Krste Misirkov 2, 1000 Skopje, Macedonia; 2 Institute of Physics, Faculty of Natural Sciences and Mathematics, Ss. Cyril and Methodius University, Arhimedova 3, 1000 Skopje, Macedonia; 3 Institute of Physics & Astronomy, University of Potsdam, D-14776 Potsdam, Germany; 4 Asia Pacific Center for Theoretical Physics, Pohang 37673, Republic of Korea; 5 Faculty of Computer Science and Engineering, Ss. Cyril and Methodius University, 1000 Skopje, Macedonia

**Keywords:** complex networks, random walks, stochastic resetting, node centrality

## Abstract

We introduce a refined way to diffusely explore complex networks with stochastic resetting where the resetting site is derived from node centrality measures. This approach differs from previous ones, since it not only allows the random walker with a certain probability to jump from the current node to a deliberately chosen resetting node, rather it enables the walker to jump to the node that can reach all other nodes faster. Following this strategy, we consider the resetting site to be the geometric center, the node that minimizes the average travel time to all the other nodes. Using the established Markov chain theory, we calculate the Global Mean First Passage Time (GMFPT) to determine the search performance of the random walk with resetting for different resetting node candidates individually. Furthermore, we compare which nodes are better resetting node sites by comparing the GMFPT for each node. We study this approach for different topologies of generic and real-life networks. We show that, for directed networks extracted for real-life relationships, this centrality focused resetting can improve the search to a greater extent than for the generated undirected networks. This resetting to the center advocated here can minimize the average travel time to all other nodes in real networks as well. We also present a relationship between the longest shortest path (the diameter), the average node degree and the GMFPT when the starting node is the center. We show that, for undirected scale-free networks, stochastic resetting is effective only for networks that are extremely sparse with tree-like structures as they have larger diameters and smaller average node degrees. For directed networks, the resetting is beneficial even for networks that have loops. The numerical results are confirmed by analytic solutions. Our study demonstrates that the proposed random walk approach with resetting based on centrality measures reduces the memoryless search time for targets in the examined network topologies.

## 1. Introduction

Random walks on complex networks as models of stochastic exploration of such topologies have been applied to study various phenomena such as search and its efficiency [1,2], network structure determination [3,4], link prediction in graphs [5], or ranking of web pages [6], among others. Various navigation strategies have been proposed to improve the search efficiency, e.g., biasing [7], use of memory [8] and long-range hops [9], or stochastic resetting [10]. Here, we combine random walk network exploration with the concept of stochastic resetting. Resetting, when a stochastic process is returned to its initial position at random times, is a natural mechanism in many search processes, such as human behavior of finding resources [11], foraging [12], population dynamics [13], etc. The number of scientific papers on resetting in diffusion and random search processes [14,15,16,17,18,19,20,21,22,23], as well as in random searches on complex networks [10,24,25,26,27,28,29], is increasing rapidly, and it has been a trending topic in non-equilibrium statistical physics in the last decade. Despite the recent increase in the interest of random walk exploration of networks with stochastic resetting, we find that, to our knowledge, previous literature does not address which node is more favorable to be set as the reset node. In this work, we aim to present a prospective way of choosing the resetting node based on node centrality measures. Using network science, we analyze why some nodes have intrinsically more control of information spread than other nodes. We establish that these nodes are the centers, essential building blocks that are modular origins of most networks [30]. We show that these center nodes, when used as resetting node candidates, have a particular minimization effect on the expected number of steps for the first arrival to a given target node—the Mean First Passage Time (MFPT). This centrality-based resetting was motivated by operations research literature [31,32], where the most common problems involve the selection of an optimal site in a network that minimizes the travel time from the facility to any other node in the network. These problems are mainly narrowed down to min-max location problems, which end up picking the site based on a global metric—the geometric center, the set of nodes that minimize the maximum distance to all nodes. We explore this property of nodes known in graph theory literature as eccentricity [33]. This effect of eccentricity has been studied on biological networks [30,34], social networks [35], and computer networks [36]. In biological networks, for instance a protein signaling network, the eccentricity can be interpreted as the likelihood of a protein to be functionally linked to all other proteins in the network. Thus, a protein with high eccentricity, compared to the average eccentricity of the network, will be more easily influenced by the activity of other proteins (the protein is subject to a more stringent or complex regulation) or conversely could easily influence several other proteins. In contrast, a low eccentricity, compared to the average eccentricity of the network, could indicate a marginal functional role. We explore this way of resetting of the random walk on several distinct network structures, where from undirected networks we mainly explore complex networks and special types of graphs (such as barbell, lollipop and balanced trees). Although for special graphs that are tree-like, the center is located trivially, for networks with distinct degree distributions, the diffusion and search properties of a random walker vary considerably. With the use of the already established discrete-time Markov chain theory [37], we derive the theoretical model for the fundamental matrix for the random walk with stochastic resetting, from which we obtain the MFPT for each pair of nodes. To evaluate our theory that the center node plays a crucial role in the utilization and information control of the network, we calculate the Global Mean First Passage Time (GMFPT) [38] from the resetting node to all the other nodes. Based on these findings, we use this measure to compare different resetting node candidates derived from other node centralities, such as degree centrality, eccentricity, or closeness centrality. We then compare both the theoretical and numerical results for all resetting node candidates and conclude that resetting to one of the centers almost surely reduces the searching ability of the resetting node to all the other nodes.

## 2. Materials and Methods

### 2.1. Random Walk on Networks with Stochastic Resetting

We denote by A the adjacency matrix with entries $a_{ij}$, which are unity when the nodes *i* and *j* are connected, and zero otherwise. The Markov transition matrix P, which is assigned with a random walk on the network, has elements $p_{ij}$, which represent the probability to jump from the node *i* to node *j*. For an ordinary random walk, the algebra $k_{i}$ denotes the number of neighbors of the node *i*, and one has $p_{ij}=\left(1/k_{i}\right)a_{ij}$. Thus, the transition matrix is positive, but not symmetric in general.

In the case of a random walk with stochastic resetting, we denote the index of the fixed resetting node with *r*, which in general does not need to be the starting node for the walk. We will assume that the probability $\gamma_{0}$ for resetting to the initial node is equal from all nodes regardless of where the walker is located. Then, for a given ordinary random walk (without resetting) determined with the transition matrix with entries $p_{ij}$, one can determine a related transition matrix for the random walk with resetting with entries $p_{ij}^{*}=\left(1-\gamma_{0}\right)p_{ij}$ for all *j* nodes that are different from *r*. If the resetting node *r* is a nearest neighbor to the current node *i*, besides the probability $\left(1-\gamma_{0}\right)p_{ir}$ for an ordinary jump, there is also a probability $\gamma_{0}$ to make a resetting jump. So, the entries in the transition matrix associated to the random walk with resetting are

(1) $$p_{ij}^{*}=\left(1-\gamma_{0}\right)p_{ij}+\delta_{jr}\gamma_{0},$$

where we have used the Kronecker delta symbol $\delta_{jr}$ to account for the cases when the resetting node *r* is a nearest neighbor of the current node *i*. In this way, jumps to the resetting node from its neighbors could be the result of an ordinary walk without resetting or a result of the resetting. The transition matrix $P^{*}$ can be more compactly written by using a diagonal matrix Γ with elements $\gamma_{ii}=1-\gamma_{0}$ and a matrix G with all columns equal to zero except the *r*th one with elements $g_{ir}=\gamma_{0}$. To avoid adding resetting from the resetting node to itself, we set the element $g_{rr}=0$. Then, one has

(2) $$P_{r}^{*}=\Gamma P+G.$$

This matrix is used as a transition matrix in the theory for the FPT based on Markov chains.

### 2.2. Mean First Passage Time

We present in this section the key steps needed to calculate the MFPT of a random walk with resetting, between pairs of nodes in a given network. The approach pursued here is based on Markov chain theory. The background for this and another approach based on generating functions can be found in the literature [2,8,37,39]. For a strongly connected graph with jump probabilities summarized in the transition matrix $P_{r}^{*}$, one first determines the row eigenvector $w_{r}=w_{r}P_{r}^{*}$ that corresponds to the largest eigenvalue. The term $w_{j}$ of the eigenvector $w_{r}$ represents the probability that the walker will be at node *j* at infinite time. Thus, this vector contains the stationary occupation probabilities or frequency of visits of nodes by a perpetual random walk. Next, one constructs a square matrix $W_{r}$ with identical rows consisting of the vectors $w_{r}$ stacked one on top of another. The respective fundamental matrix for a random walk without memory on a complex network is then given by

(3) $$Z={\left(I-P_{r}^{*}+W_{r}\right)}^{-1},$$

where I is the identity matrix of the same size as $P_{r}^{*}$. One can construct a matrix M with elements $m_{i,j}$, that correspond to the MFPT between the starting node *i* and the target node *j*. Its elements are obtained using the fundamental matrix entries, $z_{i,j}$, from the relationship

(4) $$m_{i,j}=\frac{z_{j,j}-z_{i,j}}{w_{j}}.$$

As a measure of the searching ability of the random walk with resetting, we use the average associated with a specific initial (reset) node *r* with target node *j* running over all sets of nodes, the GMFPT

(5) $$g_{r}=\frac{1}{N}\sum_{j=1}^{N}m_{r,j}.$$

### 2.3. Node Centrality Approach

Network analysis has become one of the most prominent branches of contemporary complex systems theory [40,41,42]. Almost all dynamic systems are composed of interconnected actors, and their representation in many cases can be best portrayed in terms of networks. As a result, in recent years, even highly fashionable topics such as Machine Learning have turned to this concept of representing problems as networks or graphs, as they are commonly referred to in the community [43,44]. Any mapping of structured relational data onto a graph yields higher performance in numerous domains of Machine Learning [45]. Networks in many cases very clearly convey the complex information of the systems they tend to represent. Fundamentally, these representations are composed of two parts: nodes (actors) and edges (connections). A network representation can be rather trivial, but it can also incorporate more features. This allows users to build more sophisticated representations simply by adding meaning to the connections between actors. This can be performed, for example, by using appropriate weights on the edges or adding directionality as to which actor influences whom. Nevertheless, describing a system with networks allows researchers to afterwards explore their problem representation using different network science techniques, and unveil new aspects that were not taken cognizance of before. One inherent approach to exploring network properties is based on simple random walks, in which a random walker jumps to the neighbors of a node via the edges. Random walks are at the core of several methods and algorithms used to uncover certain properties in networks [46,47,48]. The real-life problems we tend to represent typically share this hierarchy of their components, meaning some nodes or edges are more important than others. For example, in a protein–protein network, one protein may interact with many other proteins, rendering it essential for many metabolic processes that are running off within a cell. In contrast, for example, in an airplane network, one edge may be a very crucial plane connection that associates two continents.

Hence, this concept of importance is generally a big question that scientists ask when studying networks: What cardinal properties of networks are consequences of individual nodes or edges, and how does their importance influence the structure of the network as a whole? This question appears in various fields such as the famous PageRank algorithm for ranking the importance of web pages [49], stock price correlation networks in economics [50], important accounts in social networks [51,52], forecasting which actors play a crucial role in information diffusion [53,54], gossip spreading [55], epidemics [56,57], etc. Although these fields are quite different, there is one commonality: the way in which we analyze the networks, based on the structural properties of networks as a whole or the nodes individually.

**Figure 1:**
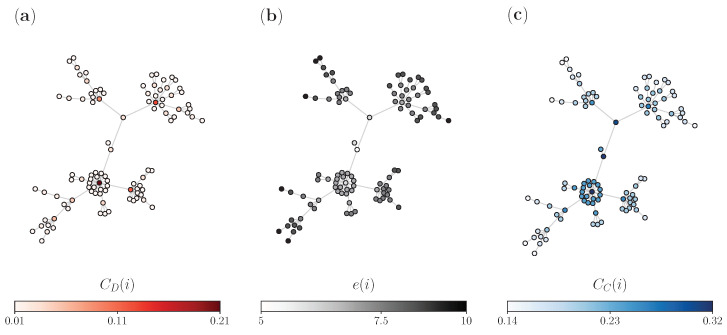
Visual representation of centrality measures on a Barabási-Albert (BA) network with *N* = 100 nodes and 2 average nearest neighbors per node, 〈k〉≈2: (a) degree centrality; (b) eccentricity; and (c) closeness centrality.

Hence, in the following part of this subsection, we will briefly introduce the notion of centrality measures of networks and how one may benefit from their assessment of node centrality importance. The objective is to display an analysis of why some nodes should be more favored as starting and resetting node candidates in our random walk model with stochastic resetting, which we introduced in the previous section. We cover basic definitions of three established centrality measures, calculate them for all nodes in a given network (Figure 1), and deduce from these results how they can help us to improve this memoryless search by resetting.

Firstly, we specify the degree centrality of node *i*,

(6) $$C_{D}\left(i\right)=\frac{\sum_{j}a_{ij}}{\left(N-1\right)}=\frac{k_{i}}{\left(N-1\right)},$$

where, as introduced before, $a_{ij}$ are the adjacency matrix elements of the network *G*, *N* is the number of nodes, and $k_{i}$ is the number of direct neighbors of node *i*. From panel (a) in Figure 1, we see that the degree centrality of nodes in undirected networks shows how well a node is directly connected (number of neighbors) and does not account for the structure of the network. Nodes in darker red are well connected, while nodes with brighter colors are nodes with few neighbors.

Secondly, we specify the eccentricity [33] of node *i*,

(7) $$e\left(i\right)=\max_{j\in V(G)}\;d\left(i,j\right),$$

where *G* is a connected network with a finite non-empty set V=V(G) of nodes together with a set E=E(G) of edges joining certain pairs of distinct nodes of *G*. The distance d(i,j) between nodes *i* and *j* is the length of the shortest path joining *i* and *j*.

The eccentricity of any node *i* intrinsically measures the maximum distance d(i,j) from *i* to any of the other nodes. Two attribute lengths that are associated with the eccentricity are the diameter and radius of a network. The former is defined as D(G), and it is the maximum eccentricity that a node can have, i.e., the longest of the shortest paths between two nodes. The latter is defined as r(G), and it is the opposite of the diameter. Hence, it is the minimum eccentricity among the vertices of *G*. Both lengths are used to distinguish two groups of nodes: center and periphery. The first is the group of nodes that are the center, defined as the set of nodes whose eccentricity equals the radius, e(i)=R(G). The second is the group of nodes that are the periphery, defined as nodes whose eccentricity equals the diameter, e(i)=D(G).

Eccentricity is a graph-theoretic metric that has been considered as a classic concept in operations research problems [31,32]. This global network measure has been proposed as a standard tool for determining whether certain functions in a network have been optimally placed. For example, when deciding on placing certain buildings in a city, we may want to take into account that those buildings should be conveniently reached, such as with fire stations or hospitals. In effect, the decision is to place them at the center, where this certain functionality will allow the facility to be within a specific range of all the other places in a network. The eccentricity is visualized in the middle panel (b) of Figure 1. We see that *i* nodes with small e(i) are near the center and are colored as white, whereas nodes with larger eccentricities are far away from the center and are fully colored in darker gray. The nodes that are colored black are the periphery of the network (eccentricity equal to the diameter).

Lastly, we specify the closeness centrality [58] of node *i*,

(8) $$C_{C}\left(i\right)=\frac{1}{\sum_{j}d\left(i,j\right)}.$$

Similarly to the eccentricity, this metric is also a global measure. It allows us to see how close an actor node is, on average, to other nodes in the network (how easily it can reach all other nodes). Again, it is defined using the shortest path d(i,j), so for each node we calculate the distance from *i* to all the other nodes. From panel (c) of Figure 1, we can see the closeness centrality, considered as the metric that describes the congruent compactness and organization of one network. From (c), we can exactly see that the nodes in darker blue are ones with higher $C_{C}$, which are nodes that can quickly interact with all other nodes of the network (well connected), while nodes in the periphery (colored in white) tend to have lower $C_{C}$ and have longer distances to all other nodes.

In the following parts, we show how these node centralities can be used to improve the random walk search strategy such that they play a key role in the selection of the starting and resetting node.

## 3. Results

In this section, we explore the potential of this centrality-based stochastic resetting on different network topologies. We present theoretical results that are derived from the calculations for the GMFPT (5), our central measure for the search efficiency, when the walker starts and resets to a given node. We also compare the theoretical model with numerical simulations for random walks with stochastic resetting with different resetting node candidates to verify the theoretical findings. It is possible that the best nodes for the proposed centrality metrics may overlap, so when we present the results, we take into account one curve for these scenarios. Firstly, we provide a comparison for undirected complex networks, namely, random, scale-free, and small-world networks. Additionally, for the scale-free complex networks, we analyze the relationship between the diameter, average node degree and the search efficiency. Secondly, we apply the same comparison to special graphs including balanced trees, lollipop graphs and barbell graphs. Lastly, we employ this approach to obtain theoretical expressions for the search efficiency for different resetting node candidates on large directed real-life networks. Here, we analyze the largest strongly connected components (LSCC) of three networks: the Arxiv HEP-PH citation graph, and two separate snapshots of the Gnutella peer-to-peer file sharing networks.

### 3.1. Complex Networks

Of the known complex network structures, we examined the three most generic topologies, the scale-free, random, and small-world networks.

Scale-free networks were generated using the Barabási-Albert (BA) model, which inherently builds a network with a power-law degree distribution. The algorithm for the BA model [59] is based on two rules, the growth and the preferential attachment rules. The first is applied when adding new nodes to the initial set of nodes. At every time step, we add a new node with *x* edges, such that each new node links to *x* different nodes already present in the network. The second rule is embodied in the probability of an added node to connect to existing nodes. This probability is taken for each node individually, and it is set to be consistent to its degree.

Random graphs were generated according to the Erdős–Rényi (ER) model, which is known in graph theory as an evolution model [60]. The algorithm for this model starts with a fixed set of *N* isolated nodes and, with a certain probability *p*, independently connects a pair of two nodes using an edge. This algorithm develops a graph that corresponds to the model by the simple successive addition of random edges. For large values of the probability *p* to connect two nodes, the generated graph will be extremely dense, and there will be edges between almost each pair of nodes.

Small-world networks were generated with the Watts–Strogatz (WS) model [61], which is an interpolation between the regular lattice and the random graph. The model creates a network with the WS topology from a regular ring lattice *N* that has *k* edges per node. It starts by randomly reconnecting nodes with other nodes with probability *p*. The algorithm in this way shrinks the diameter and stimulates the small-world property as it forms pnk/2 long-distance connections from the total number of nk/2 edges. It is interesting to point out that if we change the probability to p=0 we have a regular lattice, and for p=1 we have a random graph.

**Figure 2:**
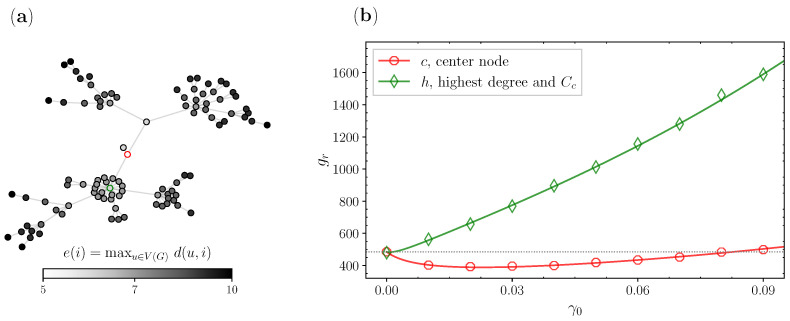
Two different resetting node candidates on a BA network: (a) BA network with eccentricity values; and (b) comparison of GMFPT $g_{r}$ (5) for the center node (red) and the node with highest degree $C_{D}$ and $C_{C}$ (green).

In Figure 2, we show one realization of centrality-based stochastic resetting on a network with a scale-free topology. In Figure 2a, we see the BA network from Figure 1, where the nodes are colored with a color map that delineates the eccentricity (7) values of each node. We can clearly see that the network is quite sparse, with a tree-like structure, and has one node with the lowest eccentricity, whose circumference is colored in red. This is the center node, which has an eccentricity of 5 (e(i)=R(G)=5) and three neighbors. Additionally, the circumference of the node with the highest degree is colored in green. This node has an eccentricity of 6 and 20 neighbors. In Figure 2b, we compare the search times when the walk starts and resets to one of the candidates.

Based on these results, we define a node candidate. A node in a network is considered a resetting site candidate if it is set as *r* in Equation (5), which denotes that it is the resetting and starting node in our theoretical and numerical calculations. We will show how different candidate nodes can improve as structural parts of a network, simply by minimizing their reachability with this stochastic resetting of the walker to a fixed resetting site. Hence, following the centrality-based approach in Figure 2, we choose the single center as our first node candidate. On the other hand, for our second node candidate, we choose the node with the highest degree (6) and closeness centrality (8) accordingly. This selection is consistent with the node centrality theory we presented in the previous Section 2.3, since we choose the node with the lowest eccentricity and the node with the highest closeness and degree centrality. The results of this comparison are shown in Figure 2b. The horizontal axis represents different resetting probabilities $\gamma_{0}$, where for each probability we analytically (lines) and numerically (markers) calculated the GMFPT $g_{r}$. Firstly, from the results, we can see that we have one curve for the node with the highest degree and closeness centrality (green) and a second curve for the center node (red). We see that resetting does not minimize $g_{r}$ when *r* is set as the hub, i.e., the walk starts and restarts to the biggest hub. At first glance, this choice makes perfect sense. The hub has this massive chunk of the network as a direct neighbor, has a low eccentricity, and has the highest closeness centrality. However, if it is chosen as the resetting site, the reachability search to all the other nodes does not minimize. On the other hand, if we look at the red curve for the center, stochastic resetting does minimize the GMFPT $g_{r}$ for around  20% from its value without resetting. The existence of some optimal resetting probability $\gamma_{0}$ can be understood by observing that when $\gamma_{0}\to 0$, one has an ordinary random walk, in which the walker can become stuck in a part of the graph far from the target, while in the other extreme, when $\gamma_{0}\to 1$, the walker could hardly go far from the resetting node. So, for some value of the resetting probability, the walker avoids being stuck, and has a better chance to explore the network. This leads to smaller search times from the center, which allow this node to exercise its potential as a geometric center in the network. We argue that this improvement is due to the fact that the center is at a radius distance of every node. Hence, enabling the random walker to jump more often to the center with a certain probability $\gamma_{0}$ allows it to reach the other nodes faster. Overall, our main takeaway from this figure and the latter reasoning lies in the idea of picking one of the centers as resetting nodes since the stochastic resetting approach makes even the center nodes “more central”. This fundamentally enhances their innate power of spreading information more rapidly to every other node in the network.

**Figure 3:**
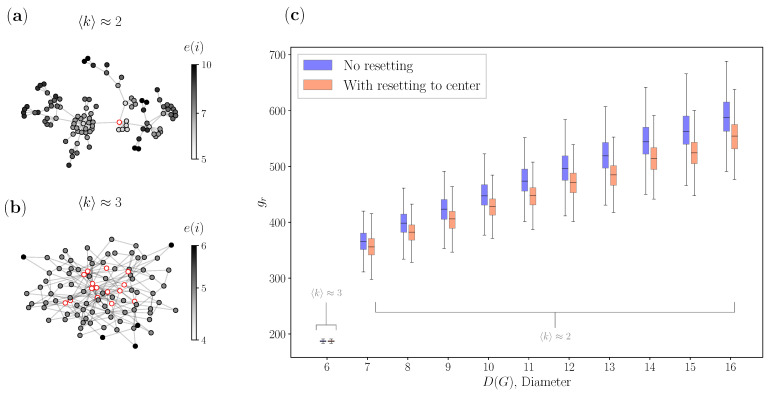
Relationship between the diameter of networks and GMFPTs $g_{r}$ (5): (a) BA network with 〈k〉≈2; (b) BA network with 〈k〉≈3; and (c) relationship between the diameter and the GMFPT $g_{r}$ for multiple generated networks.

On top of the fact that this and previous findings confirm that stochastic resetting minimizes the global search time to some extent, here we also address exactly which networks can profit from the centrality-based resetting approach. To our knowledge, this has not yet been explored. We therefore ask the following question: how can one relate network structure properties such as the connectivity and eccentricity of a given network with the gain of using a specific $\gamma_{0}$ to implement a stochastic resetting strategy on that network? In the preceding section, we introduced the concept of eccentricity and the diameter D(G), the longest shortest path. Here, we additionally introduce the connectivity, which is expressed using the average node degree 〈k〉 of the network. Having introduced both concepts, in Figure 3 we explored their relationship with the proposed centrality-based stochastic resetting. Firstly, in Figure 3a we see a BA network that has 〈k〉≈2, one center, and a maximal eccentricity (diameter) of 10. Secondly, in Figure 3b we see another BA network, but here 〈k〉≈3, such that the network has significantly more centers and has a maximal eccentricity (diameter) of 6. Although the networks have fundamentally the same BA topology, the increase of the average node degree for a notch has resulted in two completely different networks in terms of structure. The network with more edges (higher average node degree) has shrunk in diameter and has a higher density, whilst the networks with few edges are sporadic. Lastly, in Figure 3c we show the relationship between 〈k〉, D(G) and the GMFPT $g_{r}$ for a large sample of BA networks. On the horizontal axis, we have different values for the diameters D(G), where each box plot corresponds to a value of the diameter, and it is filled using $10^{3}$ independently generated networks that all have that same diameter. Blue box plots show $g_{r}$ without resetting, while the red box plots show $g_{r}$ times for resetting using the centrality-based approach for all networks. On the vertical axis, we plot $g_{r}$ for each network, where *r* is calculated for the “best” center for both approaches, with and without resetting. Going back to operations research and graph theory literature, both have addressed that one of the centers is the solution for the facility location problem [31]. If there were 10 centers as in Figure 3b, we consider the “best” center to be the one that has the smallest minimized $g_{r}$, meaning we calculate $g_{r}$ for all the centers and pick the smallest. From Figure 3c, we can directly see that BA networks can have a wide range of diameter values when the networks have a smaller average node degree. For 〈k〉≈2, we have diameter values from 7 to 16, while for 〈k〉≈3, for all of the generated networks, we only have a diameter of 6. On top of that, we clearly see that for 〈k〉≈3 (the lowest diameter value of 6), there is visibly no minimization of the GMFPT $g_{r}$ for all of the generated networks. The results are consistent with previous findings in the literature [62]: having a compact, strongly connected network has been shown to result in more beneficial nodes, since information is spreading faster. According to this finding, when the diameter of the network is small, there are many centers and the density of the network itself facilitates a faster flow of information, for which essentially no resetting is needed.

We can observe this feature in Figure 3c by looking at the blue box plots and their median values, as for diameter 6 the $g_{r}$ are naturally smaller, while for larger diameters, $g_{r}$ are increasing when *r* is chosen as the center. From comparison of the median values for $g_{r}$ with (red box plots) and without resetting (blue box plots), we conclude that resetting to one of the centers has a shrinking effect on the diameter of those networks. For example, if we look at the box plots for a diameter of 9 when we have no resetting (blue), we see that the box plots with resetting to the center (red) act as if they have a diameter of 8. This shrinking property is even more evidently seen in the drop when the diameter is 16. As mentioned before, the shrinking diameter property makes networks generally more beneficial, hence having networks with smaller diameters will most certainly improve the memory-less search we observe.

Having corroborated the benefits of resetting to one of the centers, and having established the need to analyze networks with diminished density, we continue to the analysis of the other complex networks.

**Figure 4:**
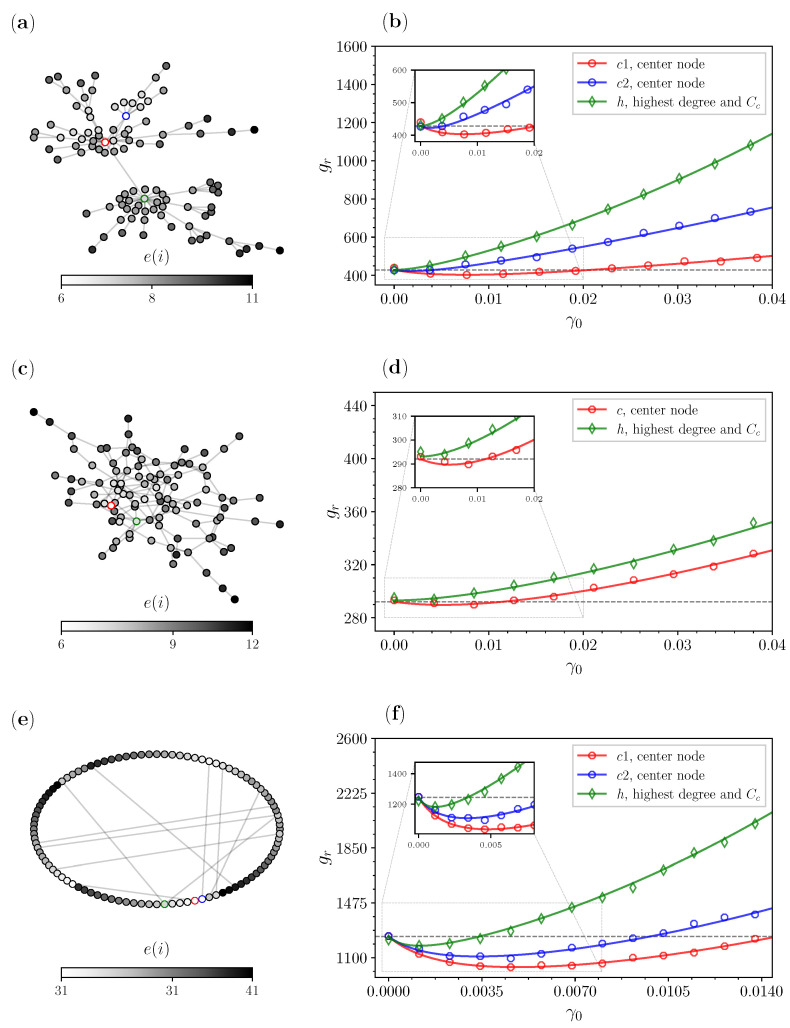
Comparison of the GMFPTs $g_{r}$ (5) for different complex networks with N=100 nodes and average node degree 〈k〉≈2: (a) BA network; (b) results for BA node candidates; (c) ER network; (d) results for ER node candidates; (e) WS network; and (f) results for WS node candidates.

In Figure 4a,b, we analyze another example of a generated BA network with 〈k〉=2, whereas this network has two centers. This means that we have two nodes that have an eccentricity equal to the radius (R(g)=6). In Figure 4b, we compare the GMFPTs $g_{r}$ when *r* is set as one of three different node candidates: $c_{1}$ (red)—the center node with higher closeness centrality (8), $c_{2}$ (blue)—the center node with lower closeness centrality, and *h* (green)—the node with the overall highest $C_{D}$ and $C_{C}$. Here again there is an overlap between the node with the highest degree and closeness centrality; therefore, we consider a single curve for this candidate. We see that, again, the approach when we reset to the center improves the GMFPT for both the center nodes. Once more, we obtain the same central result as for the scale-free networks, that resetting to the center allows the search to reach all the other nodes faster.

In Figure 4c,d, we analyze an ER network with one center and 〈k〉≈2. Networks that are generated using the ER model very rarely have a small average node degree and tend to have small diameters [59], provided that the probability *p* for connecting nodes is not too small. This is also the reason why random graphs likely have multiple centers. This characteristic has been shown in previous findings [30], which state that the ER topology has an abundance of local centers. In Figure 4d, we also compare two candidates: *c* (red)—the center, and *h* (green)—the biggest hub. We notice that the GMFPTs $g_{r}$ in the absence of resetting are smaller by a factor of almost $1^{}/2^{}$ than the ones for the BA network, whereas stochastic resetting plays no role in their minimization.

In Figure 4e,f, we analyze a WS network with two centers and 〈k〉≈2. From the left panel (e), we can see that the largest value for e(i) of any node is 41, which is a quite large diameter. The algorithm for the WS network has a tendency to stimulate compression of the diameter, but we generated a network consistent with the previously generated scale-free and random graph topologies, i.e., one that has connectivity 〈k〉≈2. The results for the node candidates for the WS network are shown in Figure 4f, where we have the same comparison as in the BA example from Figure 4b. We have three candidates for *r*: $c_{1}$ (red)—the center node with the higher closeness centrality (8), $c_{2}$ (blue)—the center node with the lower closeness centrality, and *h* (green)—the node with the overall highest $C_{D}$ and $C_{C}$. Again we see that resetting to the center improves the search to this topology as well, making it beneficial to apply to the WS models that have this configuration. Contrary to the previous two topologies, the WS model with this lower average node degree in practice produces a network with a rather homogeneous topology, meaning almost all nodes have the same degree. This equality of nodes makes this central result even more interesting.

### 3.2. Special Graphs

Of the special graph topologies [63], we examined the balanced tree, lollipop graph and the barbell graph. All of these special graphs are also “geodetic graphs” [64] since they all possess a unique shortest path between any two nodes.

Balanced tree graphs, also known as complete *b*-ary trees, are described using two parameters. The first one is the branching factor b≥2, and the second is the depth d≥1. Furthermore, the balanced *b*-tree is a rooted tree structure: a tree graph where one node is appointed as the root. The root always has *b* nearest neighbors, the other internal nodes have a degree of b+1, and the leaves have a degree of 1. The depth is also embodied by the leaf nodes, since all of them are at the same distance *d* from the root. We also consider how the different depths (sometimes called heights) can play a role in the random walk with stochastic resetting, since, as the depth increases, we have different characteristics. For example, for d=1, we have (d+1)-star graphs, and for b=2, we have the balanced binary tree.

Barbell graphs are graphs constructed by connecting two complete graphs, $K_{y_{1}}$: the two bells that are connected by a path graph, and $P_{y_{2}}$: the bridge between the two bells. Suitably, the bells have distinguished sets of nodes ($V_{left}\neq V_{right}$) where the “left bell” has $V_{left}$ nodes, and the “right bell” has $V_{right}$ nodes. The two bells are connected with $y_{2}$ bridge nodes as $V_{L}-b_{1}-b_{2}-\ldots -b_{y_{2}}-V_{right}$. Since the complete graphs that are represented by the bells are of the same order $y_{1}$, we have a symmetric adjacency matrix in the barbell: there is no difference of which is left and which is right.

Lollipop graphs are essentially barbells minus one of the two bells. They are acquired by concatenating the complete graph $K_{m_{1}}$: the bell, and the path graph, the set of pendant $P_{m_{2}}$ nodes. These graphs have a total of $N=m_{1}+m_{2}$ nodes and a structure of $K_{m_{1}}-p_{1}-p_{2}-\ldots -p_{m_{2}}$, where the pendant node $p_{m_{2}}$ is the only leaf.

**Figure 5:**
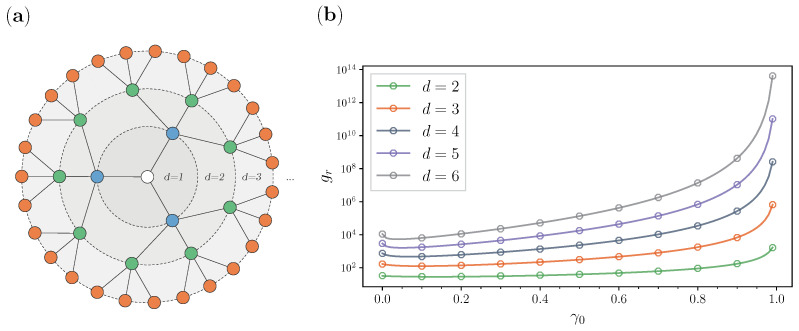
Comparison of the GMFPT $g_{r}$ (5) for different depths *d* of the balanced tree with a fixed branching factor b=3: (a) illustration of the balanced tree with depth up to d=3; and (b) analytical and numerical results for $g_{r}$ for different depths.

**Figure 6:**
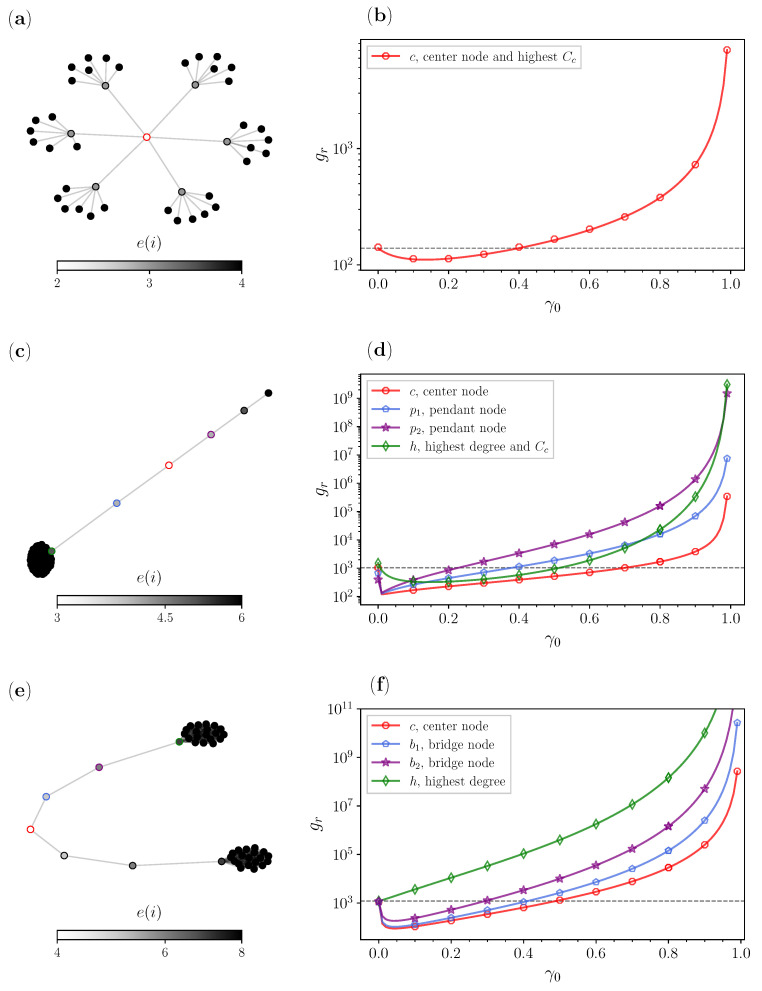
Comparison of the GMFPT $g_{r}$ (5) for different special graphs: (a) balanced tree with branching factor b=6 and depth d=2; (b) results for balanced tree node candidates; (c) lollipop graph with 5 pendant nodes and a bell of with 50 nodes; (d) results for lollipop graph node candidates; (e) barbell graph with 5 bridge nodes connecting the two bells of 25 nodes; and (f) results for barbell graph node candidates.

Figure 5 and Figure 6 show results for random walks with our centrality-based approach for specific configurations of the special graphs. Figure 5 is supplemented by Table 1, in which we detail the GMFPT $g_{r}$ improvement for different depths.

In Figure 5, we analyze the centrality-based stochastic resetting approach on balanced trees with the same branching factor but different depths. The branching factor is set to b=3, while we change the depth *d* from 2 to 6. Trees have a rather trivial center, which is the root from which all the branching starts (d=0). We see from Figure 5a that the depth is basically the radius, since the root is on a maximal distance of the depth to all nodes. The results for the GMFPT $g_{r}$ in Figure 5b are calculated results when *r* is set to be the root. These findings show that as the depth of the tree increases, $g_{r}$ becomes naturally larger, making the search from the root to all the other nodes more extensive. Due to these large values that we see as the depth increases, to appreciate the advantage of the central resetting strategy, we supplement this figure with Table 1. The table shows that as the depth increases, the balanced tree algorithm generates a tree with more nodes and a bigger diameter. The last column is similar to the previous comparisons for improvement, where we list the percentage of improvement for the optimal resetting probability $\gamma_{0}$ for which the GMFPT $g_{r}$ from the root to all the other target nodes is the smallest. This finding is consistent with the earlier diameter analysis for the scale-free networks from Figure 3. Hence, we again see that as the diameter increases, the stochastic resetting approach to the center improves the search substantially.

Figure 6 shows a comprehensive overview of the special graphs and a comparison of GMFPTs $g_{r}$ for the selected node candidates. In order to better understand the central resetting strategy, we supplement Figure 6 with Table 2.

In Figure 6a,b, we analyze once more a balanced tree, but this time with a different branching factor b=6 and a shallow depth of d=2. Similar to the previous tree analysis, we consider the center node as the resetting node candidate. This node also has the highest closeness centrality; therefore, we consider a single curve for this candidate. As we previously mentioned, the depth is equal to the radius; therefore, this center reaches all nodes relatively quickly. However, it is interesting to see from the last column of Table 2 that even tough the network is small and compact, we do have a decent improvement of $g_{r}$ of around 20%.

In Figure 6c,d, we analyze a lollipop graph with a bell with $m_{1}=50$ nodes and a path graph with $m_{2}=5$ nodes. Here we diverge a bit from our previous convention of picking the center nodes or the node with the highest degree, since we also consider two of the pendant nodes that are around the center *c*. These nodes are picked deliberately, as we wanted to show how their eccentricity plays a role. The node with the highest degree (green) is again the node with the highest closeness centrality. Then we have the center node (red), which has an eccentricity of 3 and is located right in the middle of the path graph. On top of these, here we also have two pendant $p_{1}$ nodes (blue), located between the center and the hub, and the $p_{2}$ (purple) node, right after the center node. Here we have a small improvement for the hub, but for the other three candidates, the improvements look similar at the beginning but diverge from one another for larger resetting probabilities $\gamma_{0}$. We can see from Table 2 that the largest improvement for $g_{r}$ occurs when we reset to the center, then for $p_{1}$, then $p_{2}$, which are all in agreement with the eccentricity values of the nodes. For the center, since it is the best node, even larger values for $\gamma_{0}$ do not worsen the results that fast, while for the pendant node behind it, right after the optimal reset rate we have a significant increase in $g_{r}$.

In Figure 6e,f, we analyze a barbell graph with bells that have $y_{1}=25$ nodes and a bridge path graph that consists of $y_{2}=5$ nodes. Similarly to the analysis for the lollipop graph, we consider the single center (red), the middle of the bridge, two bridge nodes, and the biggest hub (green). Due to the relatively small differences in the closeness centrality between the resetting node candidates examined for the barbell graph, we do not compare this metric when analyzing the GMFPTs $g_{r}$. Since the barbell has a symmetric adjacency matrix, we consider just one of the sides for the node candidate analysis. The two bridge node candidates are the ones between the center and the hub. First, the $b_{1}$ node (blue), the node right next to the center and the one with the lower eccentricity of the two bridge nodes are considered. Second, we look at the $b_{2}$ node (purple), the node that is right next to the hub. The transition for the GMFPTs $g_{r}$ follows the ranking in terms of the eccentricity, for both small and larger resetting probabilities $\gamma_{0}$. The results show that the best time with resetting occurs for the center (red), followed by the node with the next smallest eccentricity $b_{1}$, then the node $b_{2}$, and last the candidate *h*. From the last column of Table 2, we see that for node *h* the central-resetting does not achieve any improvement of the GMFPT $g_{r}$.

### 3.3. Real Networks

In the following section, we explore our centrality-based stochastic resetting strategy for directed real networks. Therefore, we need a modified Markov transition matrix P suitable for directed networks. So, for the uniform random walk, one uniformly chooses one of the outward links of a node. By denoting with $k_{i}^{out}$, we denote the number of links pointing to other neighbors of the node *i*—we have a probability of jumping to a neighbor $p_{ij}=\left(1/k_{i}^{out}\right)a_{ij}$ only if there is a link from *i* to *j*.

We consider three real directed networks that we obtained from the Stanford Large Network Dataset Collection [65]. The networks are the citation graph Cit-Hepth, and two different snapshots of the Gnutella hosts sharing network. For all three, we only considered their LSCC, which is essentially the largest sub-graph for which there exists a path from each and every one of the nodes.

The Cit-Hepth network is a high energy physics phenomenology citation graph [66] taken from the e-print arXiv. It covers all citations within a dataset of 34,546 papers with 421,578 edges. If a paper *i* cites paper *j*, the graph contains a directed edge from *i* to *j*. If a paper cites, or is cited by, a paper outside the dataset, the graph does not contain any information about this. The LSCC has a total of 7464 nodes, 116,268 edges, and a $\langle k^{out}\rangle =15.57$.

The Gnutella dataset [67,68] is a sequence of snapshots of the Gnutella peer-to-peer file sharing network. The dataset has a total of nine snapshots of the peer-to-peer file networks collected during August 2002. Each node in the networks represents a Gnutella host and edges represent connections between the hosts. We consider two snapshots, one from the 4 of August, and the other from 24 August. The former has a LSCC of 4317 nodes, 18,742 edges, and $\langle k^{out}\rangle =4.31$, while the latter has a LSCC of 6352 nodes, 22,928 edges, and a $\langle k^{out}\rangle =3.6$.

**Figure 7:**
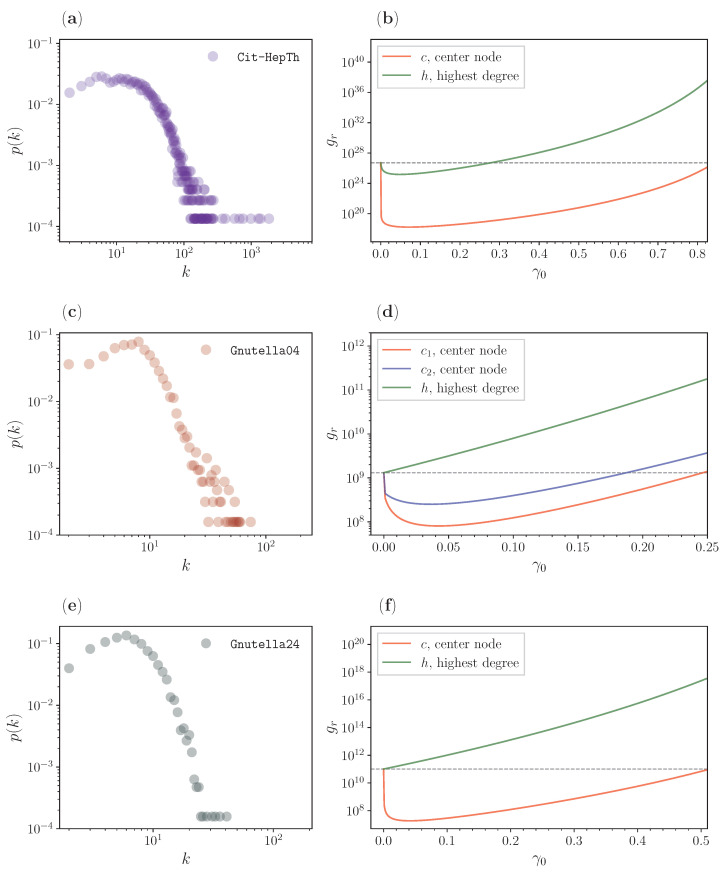
Analytical comparison of the GMFPT $g_{r}$ (5) for different resetting node candidates on the LSCC of real-life networks: (a) degree distribution of the high-energy physics theory citation network (Cit-Hepth); (b) results for different resetting node candidates of Cit-Hepth; (c) degree distribution of the Gnutella peer-to-peer network, 4 August 2002 (Gnutella04); (d) results for different resetting node candidates of Gnutella04; (e) degree distribution of the Gnutella peer-to-peer network, 24 August 2002 (Gnutella24); and (f) results for different resetting node candidates of Gnutella24.

In Figure 7, we analyze the real-life networks, where the left panels show the degree distributions that suggest a power law decay [69], and the right panels represent the analytical results for the GMFPTs $g_{r}$ for different resetting node candidates.

We show the results for the citation network in Figure 7a,b. From panel (a), we can see that the degree distribution for the network follows a power-law for higher *k* values, which reflects the scale-free property of the network. Panel (b) shows a comparison of the analytical results when *r* is either the node with the highest degree (green) or the center node (red). We can see an improvement in both cases, but for the center node, the improvement between the approach without resetting and with resetting to the center notably reaches nine orders of magnitude. This center of this citation graph is consequently a far better starting and resetting point if one has lost some reference in this citation graph. Although one might consider often going to the paper that has either the most in-citations (most cited) or the one that cites the most papers (hub: green line), this result suggests otherwise.

In Figure 7c,f, we analyze two snapshots of the Gnutella peer-to-peer network. The first snapshot is for 4 August 2002, presented in c and d. We see that, similarly to the citation graph, this network also has a degree distribution that approximately follows a power-law, reflecting some scale-free property. Here, we show a comparison of three nodes: two centers (red and blue), and the biggest hub (green). We see a similar $g_{r}$ ranking results as in the one for the candidates of Figure 4a,b. For the center, we have a significant drop, i.e., a minimization of $g_{r}$, while resetting and starting the search from the hub would not lead to an improvement of $g_{r}$. The second snapshot is from 24 August and is presented in e and f. Again, the degree distribution follows a power-law, although this power-law exponent is a bit larger [70,71] than the one for the scale-free property. We also have two candidates, the center (red) and the biggest hub (green). While there is no improvement for the hub, the resetting to the center again demonstrates the advantage of the centrality-based stochastic resetting. Analogous to the citation graph, the network topology indicates a modular organization expressed by its center. Central resetting improves the search to all the other nodes.

## 4. Conclusions

In this paper, we investigated the potential of improving the search efficiency on a network of a random walk with stochastic resetting by looking for the most plausible resetting site, which would minimize the expected time to find randomly chosen target nodes. Based on various centrality measures, we found that the node with the smallest eccentricity in the network, namely the center, is the best resetting node candidate. We confirmed this by comparing the analytical relationships based on Markov chain theory with numerical simulations of the random walk on various complex networks, special graphs, and different real networks. Choosing the center as the resetting site for directed networks results in significant reductions of the search time by a few orders of magnitude. Additionally, an interesting result is the observation that the improvement of the random search ability with resetting is better for rather sparse networks. In other words, resetting is not beneficial for denser networks, since the distribution of information is faster in such structures. Including such centralized resetting concepts in AI optimizers for random search on networks may lead to significant improvements in optimizing network searches.

The model described in this paper could be applied to studying the potential improvements of other biased walks, such as biased uniform random walks with geometric centrality, biased random walks on networks constituted by a random comb [72], and maximal-entropy bias random walks [28,73]. In future work, it could be of interest to consider measures beyond the GMFPT. From continuous, finite spaces, it is known that the density of FPTs spans many orders of magnitude between the geometry-controlled most likely FPT and the MFPT forming a long-time cutoff [74,75]. The competition between such defocused statistics with specific network structures and centrality based resetting will unveil additional features of the search strategy developed here.

## Figures and Tables

**Table 1:** Comparison of $g_{r}$ improvement for balanced trees with different depths, *d*.

*d*, Depth	*N*, No. Nodes	D(g), Diameter	$g_{r}$ Improvement ^1^
2	13	4	11.1%
3	40	6	25.4%
4	121	8	36.7%
5	364	10	45.2%
6	1093	12	51.7%

^1^ Percentage of improvement of GMFPT *g_r_* for the optimal resetting probability $\gamma_{0}$ to the center (*r*)

**Table 2:** Comparison of $g_{r}$ improvement for different special graphs.

Type of Special Graph	Resetting Node Candidate	$g_{r}$ Improvement ^1^
Balanced tree	*c*, center/highest $C_{C}$	20.27%
Lollipop	*c*, center node	88.49%
	$p_{1}$, pendant node	81.14%
	$p_{2}$, pendant node	65.68%
	*h*, highest degree/$C_{C}$	78.53%
Barbell	*c*, center	92.19%
	$b_{1}$, bridge node	90.88%
	$b_{2}$, bridge node	84.30%
	*h*, highest degree	0.00%

^1^ Percentage of improvement of GMFPT *g_r_* for optimal resetting probability $\gamma_{0}$ when resetting takes place to the resetting node candidate (*r*).

## Data Availability

No new data have been produced for this work.

## Abbreviations

The following abbreviations are used in this manuscript:

GMFPT	Global Mean First Passage Time
$g_{r}$	GMFPT
FPT	First Passage Time
MFPT	Mean First Passage Time
AI	Artificial Intelligence
LSCC	Largest strongly connected component
ER	Erdos-Rényi
WS	Watts-Strogatz
BA	Barabási-Albert
